# Clinical presentation and predictors of outcome in patients with severe acute exacerbation of chronic obstructive pulmonary disease requiring admission to intensive care unit

**DOI:** 10.1186/1471-2466-6-27

**Published:** 2006-12-19

**Authors:** Alladi Mohan, Raya Premanand, Lebaka Narayana Reddy, Mangu H Rao, Surendra K Sharma, Ranjit Kamity, Srinivas Bollineni

**Affiliations:** 1 Division of Pulmonary and Critical Care Medicine, Department of Medicine, Sri Venkateswara Institute of Medical Sciences, Tirupati 517 507, India; 2 Department of Tuberculosis and Respiratory Diseases, Sri Venkateswara Medical College, Tirupati 517 507, India; 3 Department of Anesthesiology, Sri Venkateswara Institute of Medical Sciences, Tirupati 517 507, India; 4 Division of Pulmonary and Critical Care Medicine, Department of Medicine, All India Institute of Medical Sciences, New Delhi 110 029, India

## Abstract

**Background:**

Severe acute exacerbation of chronic obstructive pulmonary disease (AE-COPD) is a common reason for emergency room (ER) visit about which little has been documented from India.

**Methods:**

Prospective study of the clinical presentation and predictors of outcome in 116 patients presenting with severe AE-COPD requiring admission to the medical intensive care unit between January 2000 and December 2004.

**Results:**

Their mean age was 62.1 ± 9.8 years. There were 102 males. Mean duration of COPD was 7.2 ± 5.8 years. All males were smokers (22.3 ± 11.2 pack years); 35.2% smoked cigarettes and 64.8% smoked bidis. All women were exposed to domestic fuel. Associated co-morbid illnesses were present in 81 patients (69.8%); 53(45.7%) had one co-morbid illness and the remaining 28 (54.3%) had two or more co-morbid illnesses. Evidence of past pulmonary tuberculosis (PTB) was present in 28.4% patients; 5 patients who also had type II diabetes mellitus had active PTB. Arterial blood gas analysis revealed respiratory failure in 40 (33.8%) patients (type I 17.5% and type II 82.5%). Invasive mechanical ventilation was required in 18 patients. Sixteen (13.7%) patients died. Stepwise multivariate logistic regression analysis revealed need for invasive ventilation (OR 45.809, 95%CI 607.46 to 3.009;p < 0.001); presence of co-morbid illness (OR 0.126, 95%CI 0.428 to 0.037;p < 0.01) and hypercapnia (OR 0.114, 95%CI 1.324 to 0.010;p < 0.05) were predictors of death.

**Conclusion:**

Co-morbid conditions and metabolic abnormalities render the diagnosis of AE-COPD difficult and also contribute to mortality. High prevalence of past PTB and active PTB in patients with AE-COPD suggests an intriguing relationship between smoking, PTB and COPD which merits further study.

## Background

Chronic obstructive pulmonary disease (COPD), a common, costly and preventable disease and is the fourth leading cause of death globally [1,2]. Internationally there is a substantial variation in death rate due to COPD possibly reflecting smoking behaviour, type and processing of tobacco, pollution, climate, and genetic factors. Given the fact that there is an increasing tendency to abuse tobacco [3-6], prevalence of COPD is expected to increase in the years to come. Acute exacerbation of COPD (AE-COPD) is a common cause of emergency room (ER) visits and is a major cause of morbidity and mortality. Following an acute exacerbation, majority of the patients experience a temporary or permanent decrease in the quality of life [7]. Moreover, more than half the patients discharged with AE-COPD often require re-admission in the subsequent six months [7]. Thus, the economic and social burden of AE-COPD are extremely high [1]. The great variability in the course of AE-COPD even in patients with similar degree of pulmonary impairment renders the prediction of the outcome in a given patient very difficult. Most studies have tried to correlate impairment in both respiratory and non-respiratory physiology with the course and progression of the AE-COPD with inconclusive results.

Though AE-COPD is a common reason for ER visits, little has been documented about this problem from India. Furthermore, even less data are available from India regarding the prevalence, precipitating factors and predictors of prognosis in patients with AE-COPD. Even from the developed world, while there are many published studies regarding the prognostic factors among patients with AE-COPD who are ambulatory, few studies have examined the prognostic factors in patients with severe AE-COPD who visit the ER and little is known regarding the long-term prognosis of patients with AE-COPD [8]. Keeping these factors in mind, the present study was designed to prospectively study the clinical presentation and predictors of outcome in patients with AE-COPD requiring admission to the intensive care unit (ICU).

## Methods

During the period January 2000 to December 2004, 914 patients were diagnosed to have COPD and were treated and followed-up from the Medicine Out-patient Department and Chest Clinic of our tertiary care referral centre. During the study period, 314 of them presented to our ER with AE-COPD. Of these 314 patients, after initial stabilisation and management in the ER, 116 were admitted to the medical ICU; 18 were discharged from the ER; and the remaining 180 were admitted to the acute medical care unit and the medical wards of the hospital. The predictors for mortality were studied in the 116 patients who were admitted to the medical ICU. Patients in whom the primary cause of ER visit was bronchiectasis, interstitial lung disease, acute severe bronchial asthma, pulmonary edema or pulmonary embolism were excluded from the study. The study was approved by the institutional ethics committee.

In all of them, pulmonary function testing was done using Morgan Transfer Test Benchmark PFT System (Morgan Scientific, Inc. Haverhill, MA, USA) at the time of the initial out-patient visit and COPD was diagnosed based on the criteria laid down by the American Thoracic Society (ATS) [8] and post-bronchodilator forced expiratory volume in the first second to forced vital capacity (FEV_1_/FVC) ratio less than or equal to 0.7 was documented in all of them confirming the presence of airflow limitation that is not fully reversible. They were all receiving a combination of various bronchodilators and anticholinergic agents.

AE-COPD was diagnosed if all of the following criteria were present at the time of ER visit: (i) recent rapid worsening of dyspnoea; (ii) increase in sputum purulence; and (iii) increase in sputum volume. Patients were eligible for only a single enrollment in this study. Hospital admissions during the study period subsequent to the index admission were not considered in the analysis.

In all of them, a detailed history was taken and a thorough physical examination was done. These details were recorded in a predesigned proforma. From the smoking history, number of "pack years" was computed in cigarette smokers from the average number of cigarettes smoked per day, one pack year being smoking of 20 cigarettes per day for one year. Since the net weight of tobacco in a bidi (150 to 240 mg) is about one-fourth that in a cigarette [9,10], in bidi smokers, "cigarette equivalent pack years" were computed. This was arrived at by dividing the "pack years" calculated on the basis of smoking bidis by four [9,10]. History of exposure to domestic fuel was recorded in female patients who were non-smokers.

At admission, in all the patients full haemogram, serum biochemistry; urine analysis were performed. Sputum and blood culture examination were performed to identify the etiological cause. Other diagnostic investigations related to the co-morbid illnesses and for monitoring the treatment were performed where they were required. Depending upon the clinical condition of the patient, bed side portable or postero-anterior view chest radiograph were performed at admission. In all the patients, 12-lead electrocardiogram (ECG) and echocardiography were performed within 24 hours of admission after initial stabilisation. Cor-pulmonale was diagnosed if there was ECG (p-pulmonale; right axis deviation; right ventricular hypertrophy) and echocardiographic evidence of right ventricular hypertrophy/dilatation. Two ml of heparinised blood sample was procured for arterial blood gas (ABG) analysis from the radial artery and was transported to the laboratory immediately for processing. ABG analysis was done using AVL Compact 2 (Radiometer, Denmark) analyzer.

Oxygen was administered through a standard dual-prong nasal cannula or face mask. When hypercapnia was a concern, oxygen was delivered through a Venturi mask (maintaining a fixed ratio of oxygen to room air). The oxygen therapy was guided by the ABG report and oxygen saturation (SaO_2_) measured using a pulse oximeter. Initially, salbutamol (as frequently as 5 mg every 15 minutes to every 8 hours) and ipratropium bromide (as frequently as 0.5 mg every 15 minutes to 0.5 mg every 8 hours) were administered through an ultrasonic nebuliser. If the aerosol therapy proved inadequate, intravenous aminophylline was administered using a constant volume infusion pump. They received injectable corticosteroids (hydrocortisone/methyl prednisolone) for 72 hours following which oral prednisolone was administered in a dosage 0.75 mg/kg body weight for a subsequent period of seven more days. Pharmacological treatment was optimized based on the clinical response. Empirical antibiotic treatment was initiated as appropriate in 104 patients (89.6%) and the antibiotic choice was further modified basing on the culture and sensitivity report.

Criteria for intubation were not standardized, and noninvasive ventilation was infrequently utilized at our hospital during the period of study. Endotracheal intubation and assisted mechanical ventilation were initiated when pharmacologic and other non-ventilatory treatments failed to reverse clinically significant respiratory failure. Indications for initiating invasive mechanical ventilation included any of the following: severe dyspnea with use of accessory muscles and paradoxical abdominal motion;. severe acidosis (pH < 7.25) and hypercapnia (PaCO_2 _> 60 mmHg); life-threatening hypoxaemia [arterial oxygen tension (PaO_2_)/inspiratory oxygen fraction (FIO_2_) < 200 mmHg)]; tachypnoea (>35 breaths/min); respiratory arrest; somnolence, impaired mental status; presence of co-morbid illness; and presence of other complications. The associated co-morbid illnesses were monitored and treated appropriately. Criteria for discharge from hospital included patients clinically and ABG wise stable for 24 hours; inhaled β2-agonist therapy is required no more frequently than every 4 hours; patient is able to eat and sleep without frequent awakening by dyspnea [1].

### Statistical Analysis

Variables following normal distribution were summarized by mean and standard deviation. The association between two categorical variables was evaluated by χ^2 ^test or Fisher's exact test as appropriate. Student's 't' test (for normally distributed variables) was used to compare the difference in mean values in the two groups for quantitative variables. To determine various predictors of death following hospitalization for AE-COPD, the analysis was performed in two stages. For this purpose the quantitative variables were categorized. Variables showing statistically significant association with the outcome (death during in-hospital stay) at p < 0.20, were considered as candidate variables for inclusion in the multivariate model. Stepwise multivariate logistic regression was performed with the potential candidate variables as the co-variates. SYSTAT version 7.0 (SPSS Inc., Chicago, USA) was used for data analysis. All the statistical tests performed were two tailed; p < 0.05 was considered as statistically significant.

## Results

The mean age of the patients was 62.1 ± 9.8 years (Table 1); there were 102 males. Their demographic parameters, smoking history, COPD staging based on pulmonary function testing done prior to the present episode of ER visit as per the Global Initiative for Chronic Obstructive Lung Disease (GOLD) staging [1] and details regarding co-morbid conditions are depicted in Table 1. Majority of the males (64.8%) smoked bidis. All women gave a history of exposure to domestic fuel such as dried cow dung cakes, wood, dried coconut shells, crop residues and grass.

Evidence of past pulmonary TB was present in 28.4% patients presenting with AE-COPD requiring admission into the medical ICU (n = 116). In five of these patients who also had type II diabetes mellitus, sputum smear-positive pulmonary TB was diagnosed at the time of presenting to the ER with AE-COPD. Three patients with type II diabetes mellitus also had diabetic ketoacidosis. On comparing the prevalence of past pulmonary tuberculosis (TB) among the 314 patients with AE-COPD (116 patients with AE-COPD who were admitted to the medical ICU, the 198 patients who presented to the ER, but who did not require admission into the medical ICU) and the remaining 600 patients who were on follow-up but who did not develop AE-COPD, it was found that patients with past pulmonary TB were more likely to suffer from AE-COPD than those who did not have pulmonary TB (61 of 314 vs. 24 of 600; χ^2 ^= 56.343, p < 0.001).

Table 2 depicts the clinical presentation of AE-COPD. All the 15 of the 116 (12.9%) patients with altered sensorium manifested one or more metabolic abnormalities [hyponatremia (n = 9); hypokalemia (n = 7); hyperbilirubinemia (n = 3); elevated transaminases (n = 12) elevated blood urea (n = 11); and elevated serum creatinine (n = 3)] or type II respiratory failure and carbon dioxide retention (n = 11). Cor-pulmonale was present in 23 (19.8%) patients.

Chest radiograph revealed infiltrates in 48 (41.4%) patients; 33 had evidence of past pulmonary TB; 5 patients were diagnosed to have sputum smear-positive pulmonary TB; in the remaining 10 patients, focal parenchymal infiltrates without air-bronchogram, suggestive of lower respiratory tract infection were present and all these 10 patients had negative sputum and blood culture. None of the five patients with AE-COPD who had active pulmonary TB (n = 5) had any symptom or sign suggestive of active TB when they last presented to the out-patient department/chest clinic for follow-up. Furthermore, they also did not have any past history of TB. Pulmonary TB was diagnosed in them only at the time of the ER visit with AE-COPD.

Laboratory abnormalities at initial presentation are shown in Table 3. ABG analysis revealed respiratory failure in 40 (33.8%) patients; 7 (17.5%) manifested type I and 33 (82.5%) patients manifested type II respiratory failure. Bacterial isolates were grown in 25 (21.6%) patients; in 11 (44%) patients, more than one pathogen was isolated. Of these isolates, *S. pneumoniae *(42.9%) and Klebsiella sp (35.7%) were the most common bacteria isolated.

There was no statistically significant difference in the clinical presentation and laboratory abnormalities between smokers of bidi and cigarette. Invasive mechanical ventilation was required in 18 (15.5%) patients. Overall, 16 (13.7%) patients died. This included patients requiring mechanical ventilation (n = 8), patients who had type II diabetes mellitus and diabetic ketoacidosis (n = 3); type II diabetes mellitus and active pulmonary TB (n = 5) at presentation. The mean duration of in-hospital stay was 6.8 ± 6.6 days.

The predictors of in-hospital death in patients with AE-COPD as per univariate sensitivity analysis and stepwise multivariate logistic regression analysis are shown in Tables 4 and 5 respectively. Type of smoking (cigarette vs. bidi) did not influence the outcome (death). Stepwise multivariate logistic regression analysis revealed need for invasive ventilation (p < 0.001); presence of co-morbid illness (p < 0.01) and hypercapnia (p < 0.05) were predictors of death.

## Discussion

Reliable epidemiological data regarding the burden of AE-COPD in the ER are lacking from India. Even less is known regarding the clinical presentation and outcome of AE-COPD in a predominantly bidi smoking population similar to the patients included in the present study. Observations from the present study indicate that patients with AE-COPD had one or more co-morbid conditions and metabolic abnormalities at presentation. High prevalence of past pulmonary TB was observed and active pulmonary TB was identified to be an important infective cause of AE-COPD.

Bidi smoking is more common in lower and middle income groups especially those residing in smaller towns, and rural areas of India as bidis are cheaper than cigarettes. Furthermore, bidi smoking is considered to cause about two to three times greater nicotine and tar inhalation than do conventional cigarettes, due to the poor combustibility of the bidi and greater puff frequency needed to keep the bidi alight [11]. All these factors may exaggerate the health risks associated with bidi smoke. The burden of tobacco use is shifting from developed to developing countries and it is generally believed that smoking habit is on the rise in India [5,12]. Therefore, the prevalence of COPD is expected to increase in the years to come and AE-COPD is likely to be an important reason for ER visits in India.

Clinical presentation of AE-COPD observed in the present study (Table 2) was similar to that reported from studies reported from other parts of the world [7,13-18]. Several causes can contribute to altered sensorium in patients with AE-COPD. These include, type II respiratory failure and carbon dioxide narcosis, metabolic abnormalities such as dyselectrolytemia, uremia and hepatic function derangement among others. As these can be corrected, an active attempt must be made to identify them when patients present to the ER with AE-COPD. This is important in developing countries like India because, majority of the patients with AE-COPD seek emergency care at primary health centres, district hospitals and general hospitals where facilities for round-the-clock laboratory monitoring are seldom available. Unless these factors, that are often correctable, are specifically sought and checked, they may be missed. Thus, these factors not only confuse the diagnosis but also contribute to mortality.

Majority of the patients in the present study had co-morbid conditions (n = 53; 45.7%) (Table 1) and presence of co-morbid factors was a predictor of death (Table 5) in these patients. Co-morbid conditions can be a confusing factor when assessing a patient with AE-COPD, as they themselves can cause respiratory symptoms [19]. Furthermore, the co-morbid conditions can trigger AE-COPD and their presence has been considered to be a predictor of poor outcome in several studies [19]. In the present study, patients who presented with AE-COPD who also had type II diabetes mellitus and diabetic ketoacidosis (n = 3); type II diabetes mellitus and active pulmonary TB (n = 5) died suggesting that complications related co-morbid conditions also contribute to the morbidity and mortality. Therefore, accurate assessment of co-morbid conditions and institution of specific treatment aimed against them should also help in reducing the mortality in patients with AE-COPD.

In the present study, compared with those who did not develop AE-COPD, past history of pulmonary TB was more frequently documented in patients presenting to the ER with AE-COPD (p < 0.001). Furthermore, 28.4% patients with AE-COPD admitted to the medical ICU had evidence of past pulmonary TB and all males among them were chronic smokers (Table 1). In a survey of 60000 men aged 20 to 50 years [20], a definite correlation between the incidence of pulmonary TB and smoking has been documented. Gajalakshmi et al [21] observed that, among urban men, the death rates from medical causes of ever smokers were double those of never smokers. Of this excess mortality among smokers, a third involved respiratory disease, chiefly TB (risk ratio ever to never smoked = 4.5) suggesting that smoking per se increased the incidence of clinical TB. It has been suggested that nicotine turns off the production of tumor necrosis factor-alpha (TNF-α) by the macrophages in the lungs, and since TNF-α is crucial for the maintenance of the latent state within macrophages, reactivation may occur rendering the patient more susceptible to the development of progressive disease from latent *M. tuberculosis *infection [22]. Treated pulmonary TB is an important cause of COPD [23] and has been reported in 41% [24] to 68% [25] patients treated for pulmonary TB. Smoking seems to increase the incidence of TB and prevalence of COPD is high where smoking is highly prevalent,. Cavitation, extensive fibrosis, bulla formation and bronchiectasis have been implicated in the genesis of COPD caused by destroyed lung due to treated pulmonary TB. Thus, in areas such as India where pulmonary TB is highly endemic and smoking is on the rise, the prevalence of COPD is expected to increase and severe AE-COPD would become a significant cause of morbidity and mortality in the ER. This intriguing relationship between smoking, pulmonary TB and COPD merits further study.

Five patients presenting with AE-COPD had type II diabetes mellitus and sputum smear-positive pulmonary TB. In the studies published from the west, there are scant references to active pulmonary TB as an infective cause of AE-COPD at the time of presentation to the ER [7,13-18,26]. This observation is particularly relevant to countries where TB is highly endemic. Patients with open TB in whom the diagnosis of TB is not considered due to low threshold of suspicion constitute a health hazard not only to the treating physicians in the ER, but also to the nursing and paramedical personnel. These observations merit further evaluation.

Lack of uniform definition of AE-COPD hampers international comparisons and the evolution of uniform diagnostic testing and treatment guidelines [19]. Furthermore, initial evaluation of a patient in the ER in the guidelines issued by several international organisations are also different [27,28]. The recently published Indian guidelines deal with AE-COPD only briefly [29]. Guidelines for the initial diagnostic evaluation of AE-COPD should facilitate differentiating AE-COPD from other conditions which can mimick it such as congestive heart failure, pneumothorax, pleural effusion, pulmonary embolism and arrhythmias. With the evolution of a consensus definition [19], these differences are likely to be resolved.

Several studies have attempted to identify the predictors of poor outcome in patients with AE-COPD [7,13-18,26]. However, there has been no such study published from India to the best of our knowledge. Acute respiratory failure is a common reason for admission into the ICU in patients with AE-COPD [7,13-18,26]. We also observed that need for mechanical ventilation was associated with a poor prognosis (Table 5). The study was carried out at our tertiary care teaching institute with facilities for invasive monitoring and assisted mechanical ventilation. These facilities are not widely available and affordable in most of the Rayalaseema area of Andhra Pradesh and majority of patients needing assisted ventilation are referred here often, late in the course of their disease. This could be the reason for reason for the high prevalence of respiratory failure in these patients. In order to cope up with the expected increase in the burden of AE-COPD, there is a pressing need for making tertiary care facilities widely available and affordable in developing countries like India.

In conclusion, in addition to the host genetic factors genetic factors, smoking behaviour, accessibility to health care and presence of co-morbid conditions contribute to morbidity and mortality due to AE-COPD. Correction of metabolic abnormalities such as dyselectrolytemia and judicious use of empirical antimicrobial treatment will also help in reducing the mortality. Large scale nationwide multicentric studies are required to clarify these issues and evolve consensus guidelines. Further research is required to clarify the association between pulmonary TB and COPD.

## Competing interests

The author(s) declare that they have no competing interests.

## Authors' contributions

AM contributed to the concept and design of study, management of patients, statistical analysis, co-ordination and preparation of the manuscript; RP contributed to the concept of the study and drafting the manuscript; LNR, RK and SB participated in patient management, and drafting of the manuscript; MHR contributed to the concept of the study and patient management; SKS contributed to critical review of the manuscript and revising the article for important intellectual content. All authors read and approved the final manuscript.

## Pre-publication history

The pre-publication history for this paper can be accessed here:



## Figures and Tables

**Table 1:** Demographic characteristics and co-morbid conditions in 116 patients with acute exacerbation of chronic obstructive pulmonary disease admitted to the medical intensive care unit

Age (years) (mean ± SD)	62.1 ± 9.8
Gender	
Male	102
Female	14
Smoking (all males)	22.3 ± 11.2 pack years
Duration of symptoms (years) (mean ± SD)	7.6 ± 5.2
COPD, GOLD stage*	
Moderate [No. (%)]	25 (21.6)
Severe [No. (%)]	55 (47.4)
Very severe [No. (%)]	36 (31.0)
*Co-morbid conditions*	
Hypertension [No. (%)]	40 (34.5)
Alcoholism [No. (%)]	38 (32.8)
Type II diabetes mellitus [No. (%)]	36 (31.0) †
Past pulmonary tuberculosis [No. (%)]	33 (28.4) ‡
Coronary artery disease [No. (%)]	12 (10.3)
Chronic renal failure [No. (%)]	10 (08.6)
Number of co-morbid illnesses	
0 [No. (%)]	35 (30.2)
1 [No. (%)]	53 (45.7)
2 [No. (%)]	18 (15.5)
3 [No. (%)]	07 (06.0)
4 [No. (%)]	03 (02.6)

GOLD = Global Initiative for Chronic Obstructive Lung Disease (reference 1)

COPD = chronic obstructive pulmonary disease

* In all patients post-bronchodilator forced expiratory volume in one second (FEV_1_)/forced vital capacity (FVC) was ≤ 0.7. Moderately severe COPD, FEV_1 _= 50 – 80% predicted; severe COPD, FEV_1 _= 30 – 50% predicted; very severe COPD = <30% predicted (reference 1)

† 3 patients had diabetic ketoacidosis and 5 patients had active pulmonary tuberculosis

‡ Clinical and radiographic evidence of past tuberculosis was present

More than one co-morbid conditions were present in several patients

**Table 2:** Clinical presentation in 116 patients with acute exacerbation of chronic obstructive pulmonary disease admitted to the medical intensive care unit

**Variable**	**%**
Symptoms	
Cough	100
Increased sputum volume	100
Increased sputum purulence	100
Recent rapid worsening of dyspnea	100
Accessory muscle use	60.3
Inability to complete a full sentence while talking	60.3
Pedal edema	19.8
Fever	29.3
Altered sensorium	12.9
Upper respiratory infection	08.6
Gastroesophageal reflux	07.8
Signs	
Wheezing	100
Respiratory rate > 24/min	94.0
Crepitations	56.0
Cyanosis	33.6
Heart rate > 100/min	25.0
Elevated JVP	12.9
Systolic BP < 90 mm Hg	03.4

JVP = jugular venous pulse

**Table 3:** Laboratory abnormalities in 116 patients with acute exacerbation of chronic obstructive pulmonary disease admitted to the medical intensive care unit

**Variable**	**%**
Polycythemia (PCV >54% in men, >49% in women)	32.8
Leukocytosis [(>12 × 10^3^/mm^3^), (>12 × 10^9^/l)]	64.7
Neutrophilia [(> 70%), (> 0.7)]	77.6
Elevated ESR (>20 mm at the end of the first hour)	64.7
Hypoalbuminemia [(< 3.5 g/dl), (< 35 g/dl)]	19.0
Hyponatremia [serum sodium < 120 meq/l, (< 120 mmol/l)]	16.4
Hypokalemia [serum potassium < 3.5 meq/l, (< 3.5 mmol/l)]	16.4
Hyperbilirubinemia [(>1.2 mg/dl), (> 20.5 μmol/l)]	06.0
Elevated transaminases [>50 IU/l]	22.4
Elevated blood urea [(>50 mg/dl), (>17.9 mmol/l)]	45.7
Elevated serum creatinine [(>1.5 mg/dl), (> 132.6 μmol/l)]	19.0

ESR = erythrocyte sedimentation rate

**Table 4:** Predictors of outcome in 116 patients with severe acute exacerbation of chronic obstructive pulmonary disease requiring admission to the intensive care unit: univariate sensitivity analysis

**Variable**	**χ**^**2**^	**p-value**
Presence of co-morbid illness	1.673	0.0196
Altered consciousness	3.65	0.056
Presence of tachycardia	9.605	0.002
Peripheral edema	1.9	0.168
Hypoalbuminemia	4.3	0.038
Elevated transaminases	4.2	0.035
Acidosis	10.257	0.001
Arterial hypoxemia	4.999	0.025
Hypercapnia	2.189	0.139
Presence of new infiltrates on the chest radiograph	5.24	0.017
Need for invasive ventilation	16.178	0.0001

**Table 5:** Predictors of death in 116 patients with severe acute exacerbation of chronic obstructive pulmonary disease requiring admission to the intensive care unit: stepwise multivariate logistic regression analysis

**Variable**	**Odds ratio**	**95% Confidence intervals**	**p-value**
Need for invasive ventilation	45.809	607.46 to 3.009	p < 0.001
Presence of co-morbid illness	0.126	0.428 to 0.037	p < 0.01
Hypercapnia	0.114	1.324 to 0.010	p < 0.05
